# Neuroprotective Effects of Stem Cells in Ischemic Stroke

**DOI:** 10.1155/2017/4653936

**Published:** 2017-07-03

**Authors:** Weilin Xu, Jingwei Zheng, Liansheng Gao, Tao Li, Jianmin Zhang, Anwen Shao

**Affiliations:** ^1^Department of Neurosurgery, Second Affiliated Hospital, School of Medicine, Zhejiang University, Hangzhou, Zhejiang, China; ^2^Brain Research Institute, Zhejiang University, Hangzhou, Zhejiang, China; ^3^Collaborative Innovation Center for Brain Science, Zhejiang University, Hangzhou, Zhejiang, China

## Abstract

Ischemic stroke, the most common subtype of stroke, has been one of the leading causes of mobility and mortality worldwide. However, it is still lacking of efficient agents. Stem cell therapy, with its vigorous advantages, has attracted researchers around the world. Numerous experimental researches in animal models of stroke have demonstrated the promising efficacy in treating ischemic stroke. The underlying mechanism involved antiapoptosis, anti-inflammation, promotion of angiogenesis and neurogenesis, formation of new neural cells and neuronal circuitry, antioxidation, and blood-brain barrier (BBB) protection. This review would focus on the types and neuroprotective actions of stem cells and its potential mechanisms for ischemic stroke.

## 1. Introduction

Stroke has been the second most common disease to cause death and disability in adults around the world [1]. Ischemic stroke, which accounts for about 87% of cases, is the most common subtype of stroke [2]. Ischemic stroke is the result of insufficient blood and oxygen supply to the brain. The cell in the central portion of the ischemic tissue, known as infarct core, is afflicted with irreversible damage, and the area around the infarct core, called penumbra, is at risk of infarction and can be reverted [3]. Various causes could contribute to this pathophysiology process, such as cerebral artery stenosis, occlusion, and rupture, and eventually induced acute cerebral blood circulation disorders. It was reported that females were less likely to suffer from ischemic stroke for both the reasons of sex steroids and biologic sex [4].

The pathological mechanism in the process of cerebral ischemia and neuroprotective effects of various drugs have been comprehensively studied throughout these years, which consists of cellular apoptosis, inflammation, oxidative stress, brain edema, and BBB interruption [5, 6]. Currently, the most common strategy used for studying the pathophysiology process and selecting potential efficacious drugs was the occlusion model of the middle cerebral artery (MCAO) in mice or rats [7]. However, treatment options to date are very limited. Stem cell therapy, with its vigorous advantages, has attracted researchers around the world.

Stem cells are defined as clonogenic cells that own the capacity to self-renew and differentiate into multiple cell lineages [8]. The application of stem cells in treating multiple diseases has been present for decades, and human stem cell transplantation therapy is now a well-established treatment for various malignant and nonmalignant hematological diseases and some autoimmune disorders [9, 10]. In the past decade, the benefits of stem cells in treating ischemic stroke have been experimentally demonstrated [11]. Low incidence of adverse effects and vast therapeutic value earn great attractions.

The basic principle of stem cell therapy of cerebral ischemia is to replace ischemic tissues in an organotypic appropriate manner. Replacement of lost neurons could rebuild the neuronal circuitry. Replacement of glial cells like astroglia or oligodendroglia could regain proper nerve conduction. Moreover, the transplantation of exogenous stem cells could also provide trophic support to tissue at risk in the penumbra surrounding the infarct area [12, 13]. Moreover, the stem cells could exert its neuroprotective effects through anti-inflammation, antiapoptosis, antioxidative, blood-brain barrier protection, promotion of angiogenesis, and promotion of neurogenesis [14, 15].

This review would focus on the types of stem cells and neuroprotective actions of stem cells and its potential mechanisms for ischemic stroke.

## 2. Stem Cell Types for Treating Ischemic Stroke

### 2.1. Exogenous Stem Cells

#### 2.1.1. Embryonic Stem Cells (ESCs)

ESCs are obtained from blastocysts in the early stage with the capacity of totipotent and unlimited self-renew. They could translate into various types of cells in the central nervous system (CNS), which makes the ESCs one of the most promising stem cells in treating ischemic stroke. The ESCs were firstly isolated and reported by Evans and Kaufman and Bremnes et al. in vivo and vitro, respectively [16, 17]. Numerous studies have demonstrated that neurons stemmed from ESCs could harmonize with the cells of receptors, which also verified the underlying efficacy of ESCs. However, some factors limited the widespread application of ESCs: (1) the availability of ESCs due to ethical concerns about the use of unwarranted embryos, (2) the risk of tumorigenicity, such as teratoma, (3) cell conservation, and (4) immune reaction after transplantation.

#### 2.1.2. Hematopoietic Stem Cells (HSCs)

HSCs could be isolated from bone marrow or umbilical blood. It has been widely studied in the treatment of ischemic stroke. Taguchi and his colleagues demonstrated that administration of CD34+ cells could enhance neovascularization in the ischemic zone and thus promote neurogenesis in mice 48 hours after ischemic stroke, and reduce the infarct area [18]. Besides, peripheral blood hematopoietic stem cell (CD34+), which was directly intracerebral implanted, was observed to differentiate into glial cells, neurons, and vascular endothelial cells. They could also enhance the angiogenesis and neurogenesis [19]. In addition, the HSCs could be used in both autologous and allogeneic transplantations without ethical problems. However, the application of HSCs owns its disadvantages of consistency of number and potency of HSCs, especially obtained from umbilical cord blood.

#### 2.1.3. Neural Stem Cells (NSCs)

Exogenous NSCs were mainly obtained from the embryo or the fetus. The NSCs have the capability to differentiate into glial and neurons, thus exert its neuroprotective effects for the patients. Besides, the NSCs have no risks of tumor tumorigenicity [20, 21]. Mack demonstrated that transplantation of exogenous NSCs could significantly improve the neurological functions with little transplantation-related toxicity [22]. However, most of the studies regarding the exogenous NSCs were restricted to experimental stroke models due to the potential ethical issues. Another severe limitation of exogenous NSCs was that only a small number of cells could survive after transplantation.

#### 2.1.4. Mesenchymal Stem Cells (MSCs)

The MSCs could be isolated from bone marrow, adipose tissue, umbilical cord blood, and peripheral blood. They could also be used both in autologous and allogeneic transplantation. Many studies have shown that transplanted MSCs could secrete cytokines and growth factors, which could enhance the process of angiogenesis and neurogenesis, and subsequently improve the neurological functions [23–26]. Besides, the MSCs were reported to reduce the cellular apoptosis by downregulating the expression of caspase-3 [27]. Moreover, autologous application could avoid immune reactions without ethical problems. All the advantages abovementioned lead to the wide study of MSCs. However, the cell culture of MSCs to generate sufficient numbers requires several weeks, which limited its use in acute phage of ischemic stroke.

#### 2.1.5. Others

Induced pluripotent stem cells (iPSCs) has been widely studied since the technique was developed by Yamanaka and his colleagues in 2006. The iPSCs were observed to differentiate into several types of neural cells and could express neuronal specific markers. Besides, the iPSCs could also reduce the infarct areas and improve neurological functions in experimental models [28, 29]. What is more, the iPSCs have no concerns regarding immune reaction, ethical issues, and the source of the stem cells. However, some critical techniques remain to be resolved before it could be extensively used, such as low efficiency of reprogramming and underlying tumorigenicity. In addition, NT2N immortal cell lines have also been reported in treating ischemic stroke.

### 2.2. Endogenous Neural Stem Cells (NSCs)

Mature neurons were once considered to lose the capability of regeneration. However, more and more studies have shown that NSCs existed in the subventricular zone (SVZ) and the subgranular zone of the dentate gyrus [30–32]. In normal conditions, the endogenous NSCs are under dormant state. They could be activated by ischemic attack and could migrate to the infarct areas to replenish the lost cells. Sharp et al. found that the NSCs in bilateral dentate gyrus increase after experimental cerebral ischemic models [33, 34]. However, the number of endogenous NSCs activated by ischemic stroke was so limited that their neuroprotective role was always unsatisfied.

## 3. Neuroprotective Properties of Stem Cells in Ischemic Stroke

The neuroprotective effects of stem cells in ischemic stroke have been widely studied, and the mechanism involved in this effect includes antiapoptosis, anti-inflammation, promotion of angiogenesis and neurogenesis, formation of new neural cells and neuronal circuitry, antioxidation, and BBB protection. The following review will particularly probe into these molecular mechanisms.

### 3.1. Replacement of Damage Tissues and Formation of New Neuronal Circuitry

The most direct way in restoring neurological functions is to replace damaged tissues with new differentiated neural cells from stem cells. The MSCs could be induced to differentiate into neural cells by epithelial growth factor and BDNF in vivo. Besides, Ishibashi and his colleagues demonstrated that the totipotent stem cells could transfer to the ischemic areas, differentiate into mature neurons, and form new neural circuity, which could thus improve the neurological functions [35]. However, some researchers doubted the efficacy of this effect. In their opinion, the stem cells differentiated into new neural cells were mainly in vivo in specific cultures. The cells transplanted to the brain may exert its neuroprotective effects in other mechanisms because only a small number of cells were tracked surrounding the ischemic areas [36, 37]. Therefore, more studies regarding the roles of stem cells in replacing damaged tissues and forming new neuronal circuitry should be launched.

### 3.2. Promotion of Angiogenesis

For treating ischemic stroke, focus should not only be placed on the regeneration of neural cells, but should also be placed on the supporting tissues, such as blood vessels. Angiogenesis was augmented after stroke. Zhang and his colleagues studied the structural changes after stroke, and they found that vascular volume was increased from 3% prior to stroke to 6% at 90 days after stroke [38]. Angiogenesis has also been observed after transplantation of stem cells surrounding infarcted areas. The main mechanisms of action were the increase of vascular endothelial growth factor (VEGF) or the level of other endogenous factors, like brain-derived neurotrophic factor and fibroblast growth factor. In addition, stem cells could also exert its role in angiogenesis by regulating the expression of Notch 1, angiopoietin-1, angiopoietin-2, and so on [39, 40].

### 3.3. Promotion of Neurogenesis

Endogenous neurogenesis, the process of self-repairing, is increased after ischemic attack. Neurogenesis is necessary for the neurological recovery for patients with cerebral ischemia [41]. Jeong and his colleagues showed that mesenchymal stem cells not only reduced the apoptosis cells but also assisted in enhancing the endogenous neurogenesis by expressing the brain-derived neurotrophic factor (BDNF) [42]. Besides, Zhao et al. found that electroacupuncture (EA) treatment could activate endogenous NSC and neurogenesis in the dentate gyrus of rats [43]. In addition, MSCs were reported to ameliorate neurological deficit if rats by modifying cerebral plasticity through neurotrophic effect and forming new synapses with host brain [44].

### 3.4. Anti-Inflammatory Effects of Stem Cells

Inflammation is a complex immune response of organisms to the injury. Under normal condition, the inflammation could help to scavenge the necrotic cells or tissues and initiate the tissue repair process [45]. However, excessive activation of immune responses is harmful to the organisms and can cause injury [46]. Stem cells had exerted its double roles in regulating inflammation by upregulating anti-inflammatory cytokines and attenuating the expression of proinflammatory cytokines. Many studies have demonstrated that stem cells could reduce the expression of IL-1*β*, IL-6, and TNF-*α* in the early stage of cerebral ischemic attack. Some researchers also suggested that the delivery of stem cells should be in the early stage as the inflammation reactions would gradually attenuate with the time going on. Besides, Zhu et al. demonstrated that human umbilical cord blood mesenchymal stem cells (hUCB-MSCs) could upregulate the expression of IL-10 and downregulate the IL-1*β*, IL-6, and TNF-*α* in the peri-ischemic brain tissues at the same time [47]. In addition, allotransplantation could suppress the immune reaction of the receptors. Vendrame and his colleagues showed that HUCBCs decreased inflammation in the brain after stroke partly by suppressing T cells [48].

### 3.5. Antiapoptotic Effects of Stem Cells

Apoptosis is one type of cell death characterized by energy dependence and programmed cell death [49]. The term “apoptosis” was first described by Kerr et al. [50]. Apoptosis is of vital importance to the normal physiological metabolism, growth, and development, keeping hemostasis by scavenging the aging or damaged cells, shaping of organs, or regulating the immune system by the removal of defective and excessive cells [51, 52]. However, uncontrolled apoptosis may result in various pathological processes of different diseases, like cancers, Alzheimer's disease, and stroke [53, 54]. The effect of antiapoptosis of stem cells for ischemic stroke has been extensively verified in experimental models. This effect may be mediated by modulating the expression of some trophins, like fibroblast growth factor, brain-derived neurotrophic factor, VEGF, glial cell line-derived factor, and nerve growth factor. Zhang and his colleagues showed that NSC transplantation could significantly reduce the number of apoptotic cells in the penumbra at 7 days by upregulating the expression of Bcl-2 [55]. Besides, Zhu and his colleagues also verified the antiapoptotic effect of hUCB-MSC for ischemic stroke around the ischemic region [47]. In addition, Li demonstrated that BMSCs transplantation could upregulate Livin protein, downregulating caspase-3 protein, thus reducing the apoptosis of neural cells [56]. The antiapoptotic effect was associated with the neurological recovery in experimental models [57, 58].

### 3.6. Others

Except for the common mechanisms abovementioned, several studies have also suggested other effects of stem cells in ischemic stroke. Tang and his colleagues showed that MSCs could protect BBB integrity by downregulation of aquaporin-4 expression via p38 signaling pathway after ischemic attack [59, 60]. Borlongan et al. found that the rats that received bone marrow stromal cells transplantation were more likely and earlier to restore the BBB and CBF [61]. Besides, Calió and his colleagues also demonstrated that transplantation of bone marrow MSCs could significantly decrease oxidative stress [62].

## 4. Some Issues in Controversy

### 4.1. Optimum Timing of Treatment

Stem cells therapy has been shown to be efficacious in treating ischemic stroke. However, the optimum timing for cells delivery has not been determined. The patients could benefit from early delivery of stem cells if the aim was to activate endogenous repair mechanism and inhibit apoptosis as this process mainly occurred during the first weeks after stroke. Park and his colleagues found that the active substances secreted by the transplanted cells exerted their neuroprotective effects only in the early 3–7 days postischemia. After that, the stem cells would keep silence functionally [63]. The results from de Vasconcelos Dos Santos et al. also suggested that transplantation of bone marrow MSCs was observed to exert its function in the first week with utmost efficacy on the first day [64]. However, patients would benefit from a later timescale if the aim of transplantation was to directly replace the infarcted tissues and rebuild new neuronal circuitry as the oxidative stress and inflammation have faded away. The optimum timing of stem cell delivery is in controversy all the time. Several studies in comparison of delivery in the acute stage and chronic stage have gained conflicting results [65, 66]. So, more studies regarding the optimum timing of treatment are warranted.

### 4.2. Cell Numbers to Be Given

The cell number to be given has not been determined. It varied according to different types of stem cells, routes of delivery, and the characteristics of the patients. Excessive amounts of cells could increase the risks of tumorigenicity and thrombosis of blood vessels. No therapeutic effect could be achieved with too small number of cells. There were few clinical studies regarding the number to be given currently. Further researches should focus on this issue before stem cell therapy could be extensively clinically used.

### 4.3. Optimum Routes of Delivery

The route of stem cells delivery varied across different studies, including intracerebral implantation, intravenous route, and intra-arterial route [67]. Intracerebral implantation could directly accumulate stem cells in the infarct areas and achieve more vigorous neuroprotective effects for the patients with ischemic stroke. However, the disadvantages of intracerebral implantation are obvious that invasive operations would inevitably disrupt normal brain tissues, which could do more damages to patients [68]. The intravenous route is the easiest and safest way for the treatment and do little secondary injury to patients [69]. However, only a small number of cells could reach the brain, and thrombosis may occur after intravenous delivery of stem cells. The intra-arterial route could delivery stem cells more directly than the intravenous route and do less injury to the patients than intracerebral implantation. However, the occlusion of the artery may affect the delivery of stem cells with the route of intracarotid administration [24]. The best route of delivery has not been determined. The decision regarding the route of delivery should take several issues into account, such as safety, practicality, cell type, and the aim of treatment.

### 4.4. The Safety of Stem Cells

As numerous studies have demonstrated the efficacy of stem cells in treating ischemic stroke, some issues regarding the safety could not be ignored. A rare but serious side effect of stem cell treatment was its potential of tumorigenicity [23]. Miura and his colleagues found that murine bone marrow-derived mesenchymal stem cells (BMMSCs), after numerous passages, obtained unlimited population doublings and proceeded to a malignant transformation state, resulting in fibrosarcoma formation in vivo. Its potential mechanism involved accumulated chromosomal abnormalities, gradual elevation in telomerase activity, and increased c-Myc expression [70]. Besides, the mechanism of extracting, preparation and administration of the cells could also cause some risks of diseases, such as immune reactions and vascular thrombosis [71]. A meta-analysis regarding the safety of stem cell therapy was performed by Jeong et al. in 2014, the results of the pooled safety analysis showed that the incidence rates of death, seizure, and infection were 13%, 15%, and 15%, respectively [72]. So, the trials in the future should not only focus on the efficacy but also on the reduction of the incidence of adverse effects.

## 5. Conclusions and Perspective

Overall, numerous experimental researches in animal models of stroke have demonstrated the promising efficacy effects of varied types of exogenous and endogenous stem cells in treating ischemic stroke. The underlying mechanism involved antiapoptosis, anti-inflammation, promotion of angiogenesis and neurogenesis, formation of new neural cells and neuronal circuitry, antioxidation, and BBB protection. Significant efficacy with low incidence of adverse effects indicates the vast potential therapeutic value of stem cells in the treatment of ischemic stroke. However, some important issues, such as optimum timing of treatment, dosage, optimum routes, and some rare but serious adverse effects, have not been resolved. Further studies, both preclinical and clinical studies are warranted for an effective, feasible, and safe cell-based therapy.
